# Assessing the Impact of Dermatophytosis on Quality of Life Using the Dermatology Life Quality Index: A Real-World Cross-Sectional Study From India

**DOI:** 10.7759/cureus.106253

**Published:** 2026-04-01

**Authors:** Priyanka Singh, Manjunath Shenoy, Mukesh Girdhar, Shrichand G Parasramani, Anupam Das, Pallavi S Mishra, Dhiraj Dhoot, Saiprasad Patil

**Affiliations:** 1 Dermatology, Younique Skin Clinic, New Delhi, IND; 2 Dermatology, Yenapoye Medical College, Mangalore, IND; 3 Dermatology, Max Super Specialty Hospital, Patparganj, New Delhi, IND; 4 Dermatology, Anisha Clinic, Mumbai, IND; 5 Dermatology, KPC Medical College and Hospital, Kolkata, IND; 6 Global Medical Affairs, Glenmark Pharmaceuticals Ltd., Mumbai, IND

**Keywords:** chronic dermatophytosis, dermatology life quality index, from india, quality of life (qol), recalcitrant disease, recurrent dermatophytosis, super-bioavailable itraconazole, superficial fungal infections, topical antifungal

## Abstract

Background and objective: Dermatophytosis has emerged as a chronic, recurrent, and high-burden dermatosis in India, with increasing reports of extensive involvement and treatment difficulty. Although clinical assessment is routinely performed, patient-reported quality of life (QoL) evaluation is inconsistently integrated into practice. This multicentric real-world study aimed to assess the QoL burden associated with dermatophytosis using the Dermatology Life Quality Index (DLQI).

Methods: A cross-sectional study was conducted between April and October 2025 involving clinically diagnosed dermatophytosis patients attending the outpatient department from 325 centres across India with Independent Ethics Committee approval. The primary endpoint of the study was to assess DLQI in these patients, whereas secondary endpoints included evaluation of the association between DLQI scores and demographic, clinical, occupational, comorbidity-related, and treatment-related variables.

Results: We analysed the data of 2776 patients who met the inclusion criteria, highlighting the predominance of males (57%). The median DLQI was 12 (interquartile range (IQR) 8-17), indicating a very large impact of dermatophytosis on daily life. The QoL impairment increased progressively with disease extent, demonstrating a clear severity impact correlation relationship between lesion burden and DLQI (p<0.001). The DLQI differed significantly across age groups (p<0.001) and was markedly higher in patients with facial (p<0.001) and trunk/back involvement (p<0.001). Occupations associated with high sweat and friction exposure showed the greatest impairment (p=0.003). Systemic comorbidities, including obesity, diabetes, hepatic, cardiac, and renal disorders, were consistently associated with worse DLQI (all p<0.001). Treatment regimens significantly influenced patient-reported outcomes (p<0.001), with super-bioavailable(SBA) itraconazole combined with topical therapy demonstrating the most favorable QoL profile.

Conclusion: The QoL impairment in these patients is most pronounced with facial involvement, higher lesion counts, high sweat/friction occupations, and systemic comorbidities. Notably, treatment with SBA itraconazole in combination with topical antifungal therapy was associated with lower DLQI. These findings emphasize the importance of individualized management, prioritizing patients with visible/extensive disease and medical comorbidities for more intensive treatment and supportive counseling.

## Introduction

Dermatophytosis comprises superficial fungal infections caused by keratinophilic dermatophytes affecting the skin, hair, and nails, most commonly presenting as tinea corporis, tinea cruris, tinea pedis, tinea manuum, and onychomycosis [1]. In India, clinical patterns have shifted toward chronicity, recurrence, extensive involvement, and treatment difficulty, prompting descriptions of an 'epidemic-like' situation in routine dermatology [2].

Multiple drivers contribute to the current scenario. Environmental and behavioral factors sustain transmission, while treatment-related practices, particularly self-medication and use of topical antifungal corticosteroid combinations, promote atypical presentations, delayed diagnosis, and persistent infection [3]. In parallel, the emergence and spread of Trichophyton indotineae (approximately 70% to 80% of dermatophyte isolates in several regions, although exact population prevalence remains uncertain due to limited surveillance and diagnostic capacity), a highly transmissible species associated with antifungal resistance and recalcitrant disease, has further complicated management and highlighted dermatophytosis as a high-burden condition. [4].

The impact of dermatophytosis extends beyond visible lesions. Persistent pruritus, scaling, and discomfort can impair sleep, productivity, and social functioning, especially in recurrent or prolonged disease [2]. As such, consequences are not fully captured by morphology, and patient-reported outcome measures (PROMs) are increasingly used to quantify symptom burden and functional limitation from the patient’s perspective [5].

The Dermatology Life Quality Index (DLQI) is widely used to assess dermatology-related quality of life (QoL) across domains such as symptoms and feelings, daily activities, leisure, work/school performance, personal relationships, and treatment burden [6]. The DLQI scores are interpreted using standardized bands: 0-1 denotes no effect; 2-5 a small effect; 6-10 a moderate effect; 11-20 a very large effect; 21-30 an extremely large effect [7]. This structured system supports clinically meaningful reporting and facilitates QoL comparison across studies and populations.

Despite increasing recognition of dermatophytosis as a chronic and recurrent condition, systematic assessment of patient-reported QoL remains uncommon in Indian dermatology practice. Clinical evaluation in many outpatient settings still prioritizes morphology and mycological confirmation, with limited documentation of patient-perceived disease impact [5]. Where QoL data exist, Indian studies have largely been single-center or focused on select subgroups, limiting generalizability to broader real-world populations [8,9]. Evidence relating demographic characteristics and routine treatment patterns to QoL impairment is similarly scarce.

Given heterogeneity in disease distribution, exposures, comorbidities, and treatment approaches, multicentric real-world data are needed to characterize QoL burden and support integration of patient-reported outcomes into routine care. Accordingly, the present retrospective observational study evaluated QoL in adults with dermatophytosis using the DLQI [6] and examined its association with demographic characteristics in Indian outpatient practice.

## Materials and methods

This retrospective, observational, multicentric study was conducted between April and October 2025 using medical records of adult patients diagnosed with dermatophytosis across 325 dermatology outpatient settings in India. The study was performed in accordance with the principles of the Declaration of Helsinki, the International Council for Harmonisation-Good Clinical Practice (ICH-GCP) guidelines, and the New Drugs and Clinical Trials Rules, 2019, Government of India. The study protocol and data collection instruments were reviewed and approved by an Independent Ethics Committee, the Good Society for Ethical Research, prior to study initiation (approval no. GSER/2025/BMR/CL/062). All patient data were anonymized before analysis to maintain confidentiality.

Medical records of patients aged 18 years and above with a clinical diagnosis of dermatophytosis were included for analysis. Clinical and demographic information was collected using a predesigned structured proforma and included age, sex, occupation, dermatophytosis subtype, lesion location and lesion count, duration of disease, past and family history of dermatophytosis, presence of comorbid conditions, treatment regimen, duration of antifungal therapy, and DLQI scores. The QoL was evaluated using the DLQI questionnaire, which measures the impact of dermatological disease across symptoms, daily activities, leisure, work or school, personal relationships, and treatment-related domains [6].

The primary endpoint of the study was the assessment of overall DLQI in dermatophytosis. Secondary endpoints included evaluation of DLQI in relation to demographic variables, dermatophytosis subtype, lesion location, lesion burden, disease duration, comorbidity status, occupational category, and treatment pattern.

Statistical analysis was performed on complete-case data for core analytic variables. Continuous variables were assessed for normality using the Shapiro-Wilk test and were found to be non-normally distributed. Accordingly, data were summarized as medians with interquartile ranges (IQRs) and analyzed using non-parametric statistical methods. Comparisons of DLQI scores between two independent groups were conducted using the Mann-Whitney U test, while comparisons across more than two groups were performed using the Kruskal-Wallis test with Bonferroni-adjusted post-hoc analyses. All statistical tests were two-sided, and a p-value of less than 0.05 was considered statistically significant.

## Results

A total of 2,776 adult patients with dermatophytosis were included in the analysis. The majority were aged 31 to 45 years, and males constituted more than half of the study population. Tinea corporis and tinea cruris were the most common clinical subtypes. Across all subtypes, the median disease duration was approximately one year, reflecting predominantly chronic disease at presentation (Table 1). Additional details about the medical records included are mentioned in Appendix A.

**Table 1:** Clinical determinants of DLQI (n = 2,776) and practice and patient factors affecting DLQI Data are presented as median (IQR). The p‑values were calculated using the Kruskal–Wallis test for comparisons involving more than two groups and the Mann–Whitney U test for two‑group comparisons. Post‑hoc pairwise comparisons were performed using Bonferroni adjustment where applicable. A p-value <0.05 was considered statistically significant. ^¥^Age (H = 35.99); ^£^subtype (H = 14.50); ^Ω^Lesion site (H = 10.83); ©Lesion count (H = 16.27); ^Ô^Occupation (H = 16.34); ^Ć^Comorbidities (H = 20.52); ^Ŧ^Treatment (H = 62.42) DLQI: Dermatology Life Quality Index; IQR: Interquartile range; SBA: Super‑bioavailable

Parameter	Category	N (%)	Median DLQI (IQR)	p-value
Overall	All patients	2776 (100%)	12 (8-17)	-
Age (in years)	18-30	670 (24.1%)	11 (8-16)	<0.001^¥^
	31-45	1171 (42.2%)	13 (8-18)	
	46-60	744 (26.8%)	13 (9.8-17)	
	>60	164 (5.9%)	10 (5-16)	
Subtype	Tinea corporis	1536 (55.3%)	13 (9-18)	0.006^£^
	Tinea cruris	1236 (44.5%)	12 (7-18)	
	Tinea pedis	226 (8.1%)	12 (10-17)	
	Onychomycosis	113 (4.1%)	12 (8-16)	
	Tinea manuum	107 (3.9%)	13 (9-18)	
Lesion site	Face (yes)	251 (14.86%)	16 (12-21)	<0.001 ^Ω^
	Trunk/back (yes)	1438 (85.14%)	14 (10-18)	
	Limbs	842 (30.32%)	13 (8-17)	
	Groin	1236 (44.53%)	13 (7-18)	
Lesion count	1	270 (10.51%)	10 (6-18.8)	<0.001^©^
	2-3	1246 (48.48%)	11 (7-16)	
	4-5	871 (33.88%)	14 (10-17)	
	>5	183 (7.12%)	17 (5-20)	
Occupation	High sweat/friction	243 (10.13%)	14 (9-18)	0.003 ^Ô^
	Sedentary indoor	944 (39.33%)	12 (6-17)	
	Domestic/non-working	550 (22.92%)	13 (7-17)	
	Mixed exposure	663 (27.63%)	12 (9-16)	
	Healthcare workers	24 (0.9%)	10 (8-10.3)	
Comorbidities	Obesity (yes)	801 (28.9%)	14 (10-20)	<0.001 ^Ć^
	Hypertension (yes)	643 (23.2%)	14 (10-18)	
	Diabetes (yes)	280 (10.1%)	15 (11-19)	
	Hepatic dysfunction (yes)	61 (2.2%)	16 (11-22)	
	Cardiac disorder (yes)	69 (2.5%)	15 (11-22)	
	Renal disorder (yes)	56 (2.0%)	17 (14-19)	
Treatment	Super-bioavailable (SBA) itraconazole + topical	1168 (42.1%)	11 (6-17)	<0.001 ^Ŧ^
	SBA itraconazole alone	1045 (37.6%)	13 (10-18)	
	Itraconazole + topical	288 (10.4%)	12 (5-17)	
	Itraconazole alone	259 (9.3%)	13 (10-18)	

Overall QoL was substantially impaired, with a median DLQI score of 12 (IQR 8-17), corresponding to a very large impact on daily life. The DLQI differed significantly across age groups (p<0.001), with the greatest impairment observed in adults aged 31 to 60 years, while no significant difference was observed between males and females (Table 1).

The QoL impairment varied by clinical subtype (p=0.006), with the highest DLQI scores observed in tinea corporis and tinea manuum. Facial and trunk/back involvement were associated with significantly higher DLQI scores compared with their absence (both p<0.001) (Table 1). Increasing lesion burden demonstrated a direct relationship with DLQI (p<0.001), with the greatest impairment observed in patients with more than five lesions (Table 1).

Occupational exposure significantly influenced QoL (p=0.003). High sweat- and friction-exposure occupations (manual laborers/skilled workers, drivers/transport workers, and service/sales/support staff) showed the highest DLQI scores. Sedentary indoor occupations (salaried/government/office workers and teachers/academics) reported lower impairment, while homemakers/unemployed and mixed-exposure groups demonstrated intermediate impairment. Healthcare workers had the lowest DLQI scores (Table 1). Systemic comorbidities were strongly associated with poorer QoL. Obesity, hypertension, diabetes mellitus, hepatic dysfunction, cardiac disorders, and renal disease were each linked to significantly higher DLQI scores (all p<0.001), indicating compounded disease burden in medically vulnerable patients (Table 1).

**Figure 1:**
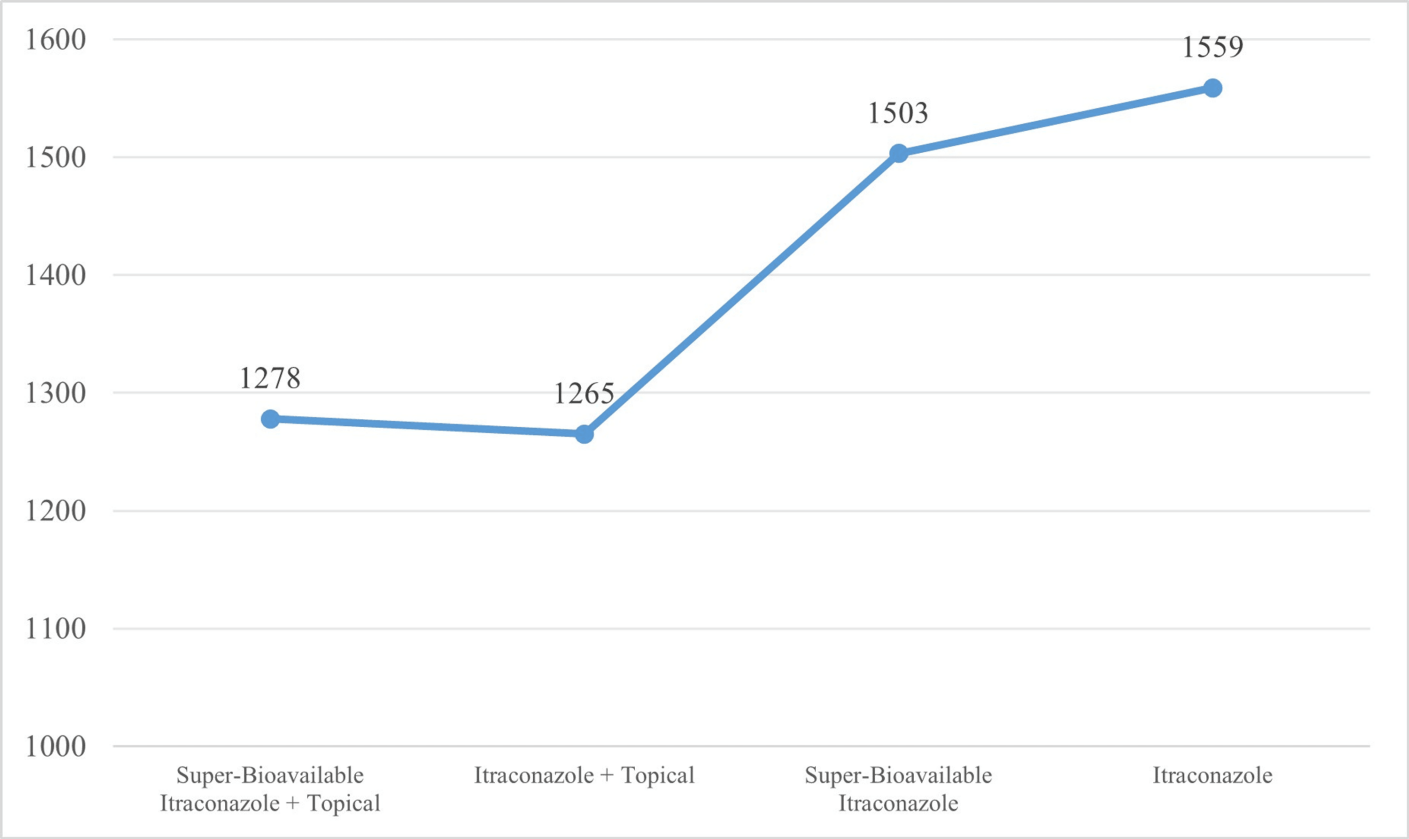
Mean rank distribution of DLQI across treatment regimens Mean rank distribution of DLQI across antifungal treatment regimens demonstrates superior QoL outcomes with SBA itraconazole plus topical therapy (p < 0.001). The p-values were calculated using the Kruskal–Wallis test. Lower mean rank = Better QoL outcome DLQI: Dermatology Life Quality Index; QoL: Quality of life; SBA: Super-bioavailable

The QoL differed significantly across treatment regimens (p<0.001). Super-bioavailable (SBA) itraconazole combined with topical therapy was associated with the lowest DLQI scores, reflecting superior patient-reported outcomes (Figure 1). Prolonged treatment duration was associated with improved DLQI outcomes in combination with SBA itraconazole regimens, whereas no significant improvement was observed with itraconazole monotherapy. Disease duration (one year) and treatment duration (1.5 years) both demonstrated significant positive associations with DLQI (p < 0.01), indicating increasing QoL impairment with chronicity and prolonged treatment requirement (Table 1).

## Discussion

In this large multicentric real-world cohort, dermatophytosis was associated with marked impairment in patient-reported QoL. The DLQI scores clustered predominantly within the very-large impact band, indicating substantial disruption of daily functioning.

The QoL impairment varied significantly with age, clinical subtype, lesion burden, anatomical site, occupational exposure, comorbidity profile, and antifungal treatment pattern. A clear dose-response relationship was demonstrated between increasing lesion count and DLQI, and facial and trunk/back involvement were associated with greater impairment. Occupations involving high sweat and friction showed the highest DLQI scores, highlighting the influence of routine environmental exposure. Comorbidities, including obesity, diabetes, hepatic, cardiac, and renal disorders, were associated with worse DLQI. Better QoL outcomes were observed with SBA itraconazole-based regimens, particularly when combined with topical therapy.

Cross-sectional cohorts from tertiary dermatology clinics in India have consistently reported that most patients with dermatophytosis experience large to extremely large DLQI impact, confirming the high day-to-day burden of disease in routine care [10]. A regional study from South Asia similarly documented that nearly 40% of patients experienced a very-large DLQI impact, with higher scores in generalized and prolonged disease [11]. These reports frame our multicentric findings within a consistent regional pattern of substantial patient-perceived morbidity.

Another observational cohort focusing on superficial dermatophytosis reported that more than 45% of patients experienced a very large DLQI impact, with the highest impairment in patients with combined facial and truncal involvement [12]. A multicenter Indian survey further documented that the majority of patients reported very-large to extremely-large DLQI impact, particularly in adults within productive age groups, emphasizing the socioeconomic relevance of disease burden [13].

Across published cohorts, disease extent consistently predicts DLQI. Multiple studies have shown significantly higher DLQI with increasing body surface area involvement, multi-site disease, generalized involvement, and relapse-prone courses [10,11]. Our demonstration of a severity impact correlation between lesion burden and DLQI directly mirrors these findings.

Age has also been identified as an important determinant, with greater impairment reported among adults in their productive years, consistent with our observation of higher DLQI in middle-aged adults [13]. Gender-based differences have been inconsistent across cohorts; while some studies observed higher DLQI in females, others reported comparable impairment between sexes [13,14]. Our finding of no significant gender difference aligns with this heterogeneity. Anatomical distribution has been shown to influence QoL, with poorer DLQI reported in patients with combined, facial, and generalized involvement [10,11]. Our observation of higher DLQI with facial and trunk/back involvement is concordant with these reports. Occupational and environmental exposure have been less frequently evaluated. Our finding that occupations associated with high sweat and friction are linked to greater DLQI adds novel real-world evidence that routine exposure can amplify symptom persistence and functional limitation.

Randomized clinical trial data have demonstrated superior clinical cure with SBA itraconazole and once-daily dosing compared with conventional itraconazole regimens [15]. The better DLQI outcomes observed in our cohort with SBA itraconazole-based regimens suggest that improved clinical control translates into better patient-reported outcomes in routine practice.

Routine DLQI screening can identify patients experiencing disproportionate impact despite similar clinical severity. Incorporating simple checks of lesion burden, site of involvement, occupational exposure, comorbidity status, and treatment regimen may help clinicians prioritize counseling, tailor therapy, and plan follow-up more effectively.

This multicentric real-world study benefits from a large sample size and use of a validated, interpretable patient-reported outcome measure (DLQI), allowing clinically meaningful assessment of QoL impairment in routine dermatophytosis care. Limitations include its retrospective design, reliance on routine clinical records with possible missing or misclassified data (as detailed in Appendix A), lack of standardized clinician-rated severity indices for severity-adjusted analyses, and absence of longitudinal follow-up to assess change in DLQI over time. Prospective longitudinal studies integrating DLQI with standardized severity measures are warranted.

## Conclusions

Dermatophytosis is associated with substantial impairment in patient-reported QoL in routine clinical practice. Lesion burden, anatomical distribution, occupational exposure, comorbid conditions, and treatment patterns significantly influence this burden. Incorporating DLQI assessment into routine care may support more individualized management and help identify patients who require targeted counseling and optimized therapeutic strategies.

## Appendices

Appendix A 

Table 2 features the additional details of the medical records of the included patients (total n = 2,776).

**Table 2:** Additional details of the medical records included for analysis

Parameters	Discrepancy	Justification
Age	Missing, not key data	Age was available for 2,749 patients; 27 records were missing age. Because age was not prespecified as a key variable for our primary endpoint, these records were retained in the cohort but excluded from age‑specific summaries, leading to an age denominator of <2,776.
Subtype	Overlapping data	A single patient presented with more than one dermatophyte subtype (e.g., tinea corporis with concurrent tinea cruris). Therefore, counts for individual subtypes overlap, and their totals exceed the number of unique patients.
Lesion site	Overlapping data	Several patients presented with lesions at multiple anatomical sites (e.g., face, trunk/back, limbs, or groin). Therefore, lesion‑site counts overlap and exceed the number of unique patients.
Lesion count	Number less than the sample analyzed	Lesion count available for 2,570 patients; the remaining 206 had documented lesion clearance (treatment continued for two weeks after complete cure), so counts were not recorded and are excluded from lesion‑count analyses
Occupation	Missing, not key data	Occupation information was documented for 2,424 patients in the source records, while 352 records lacked occupation details. These records were retained in the overall cohort (n = 2,776), but only patients with documented occupation were included in the occupation‑specific analyses. Therefore, the denominator for occupation‑related results is 2,424.
Comorbidities	Rest no comorbidities	Comorbidity data were recorded for 1,910 patients; the remaining 866 patients had no comorbidities documented and were not included in comorbidity‑specific summaries.
Treatment	Number less than the sample analyzed	Treatment was recorded for 2,760 patients; the remaining 16 had achieved lesion clearance and completed the therapy, so treatment details were not documented and were excluded from treatment‑specific summaries.
